# Characterization of fermentation parameters, volatile compounds and microbial community structure of fermented grains in the upper and lower layers during *strong-flavor Baijiu* fermentation

**DOI:** 10.1186/s40643-025-00916-2

**Published:** 2025-09-26

**Authors:** Jie Deng, Chao-Jiu He, Chun-Hui Wei, Hui-Bo Luo, Zhi-Guo Huang

**Affiliations:** 1 Liquor Making Biotechnology and Application Key Laboratory of Sichuan Province, Sichuan University of Science and Engineering, Yibin, 644000 P. R. China; 2 Key Laboratory of Brewing Molecular Engineering of China Light Industry, Beijing Technology and Business University, Beijing, 100048 China; 3 Jing Brand Co. Ltd, Daye, 4351000 P. R. China

**Keywords:** *Baijiu*, Solid-state fermentation, Microbial diversity, Volatile compounds

## Abstract

*Strong-flavor Baijiu* (SFB) is produced using a solid-state fermentation system, but the impact of stratified fermented grains on fermentation remains unclear. Therefore, in a typical distillery plant, we evaluated the physical and chemical composition, volatile compound profile, and microbial community of fermented grains in SFB both above (FG-A) and below (FG-B) the *Huangshui* line. Significant differences in fermentation parameters between FG-A and FG-B were observed after 30 days of fermentation (*P* < 0.05). Additionally, the partial least squares projection to latent structure discriminant analysis (PLS-DA) revealed distinct differences in volatile compounds between FG-B and FG-A, identifying 26 discriminant markers. The diversity of short-chain fatty acids (SCFAs) and their esters were higher in FG-B compared to FG-A. Furthermore, microbial diversity and abundance have differed significantly between the two layers of fermented grains (*P* < 0.05), included 17 differential genera. Correlation and pathway enrichment analyses indicated that the higher SCFA content in FG-B could be attributed to the greater abundance of acid-producing microorganisms compared to FG-A. This study highlights the differences between the two layers of fermented grains in SFB fermentation, offering new insights into solid-state fermentation and expanding the current understanding of the traditional SFB fermentation process.

## Graphical abstract

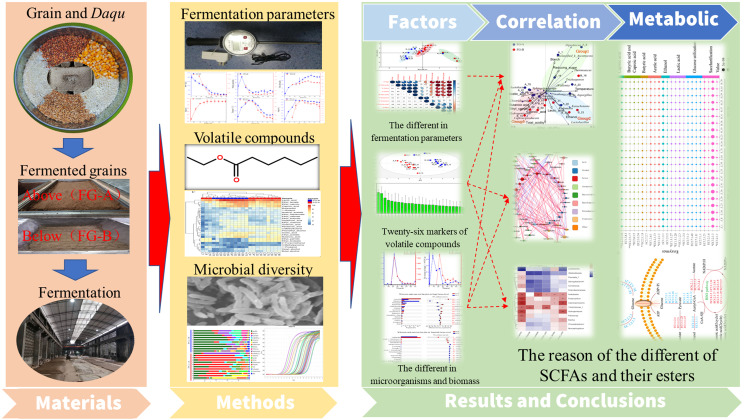

## Introduction

*Baijiu* is a traditional fermented liquor deeply embedded in Chinese culture, boasting a rich history that spans centuries. It is considered one of the world’s oldest alcoholic beverages and is typically produced through solid-state fermentation, along with whiskey, vodka, gin, brandy, and rum, is considered one of the six most popular distilled spirits (Liu and Sun 2018). There are currently twelve recognized types of *Baijiu*, each distinguished by its unique aroma characteristics and produced in various provinces across China (Zheng and Han 2016). Among these types, *Strong-flavor Baijiu* (SFB) is one of the most important types of *Baijiu*, representing approximately 50% of total *Baijiu* production in 2021. Since 2013, the annual yield of SFB has surpassed 10 million kiloliters, with sales revenue reaching billions of dollars (Liu and Sun 2018). The most renowned production sites for SFB in China include Yibin, Luzhou, and Suqian, among others.

**Fig. 1:**
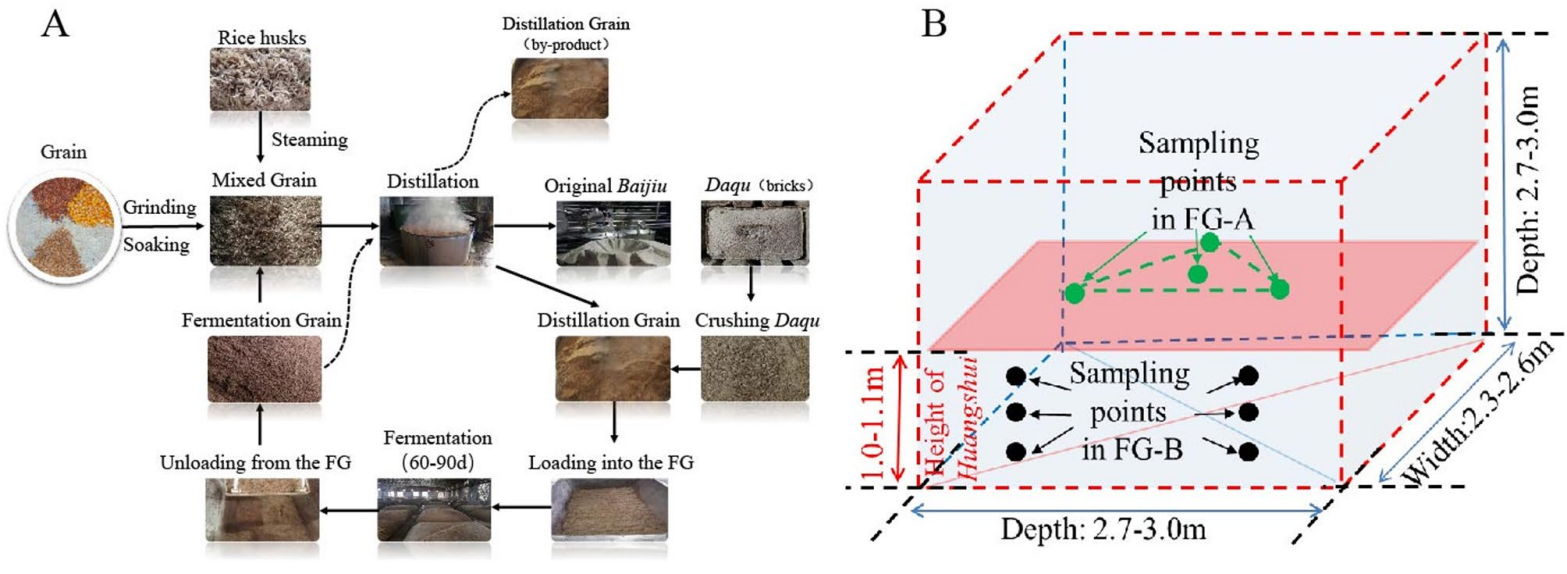
Production process of strong-flavor *Baijiu* (**A**). Sampling points in mud cellar (**B**). Black circles (●) indicate fermented grains sampling sites below (FG-B) the *Huangshui* line, green circles () represent fermented grains sampling sites above below (FG-A) the *Huangshui* line; the red rectangle represents the final liquid level of *Huangshui*; the height of *Huangshui* line was approximately 1.2–1.3 m in the winery in which the experiment was conducted

SFB is typically made from sorghum alone or a mixture of grains, including corn, rice, wheat, sticky rice, and sorghum. Ethyl caproate, a key aroma compound in SFB, show imparts fruity, floral, and sweet notes (Niu et al. 2020). Other important short-chain fatty acid esters (SCFAEs) such as ethyl acetate, ethyl butyrate, and ethyl lactate are also crucial for the aroma of SFB (Ren et al. 2024). The fermentation process begins with a starter culture called *Medium Temperature Daqu*, a complex starting material used for SFB production, contains microorganisms, enzymes, and volatile compounds, which is mixed with grains to form fermented grains (FG) (Xu et al. 2019; Yan et al. 2019). The FG mixture is then placed in fermentation vessels within an underground cuboid fermentation pit mud (FPM) that measures 2.5–2.8 m in height, 2.8–3.2 m in length, and 2.3–2.6 m in width. After sealing the pit with mud, anaerobic fermentation is carried out for approximately 60–70 days at temperatures ranging from 20 to 34 °C (Wang et al. 2020). *Baijiu* is distilled from the FG, and while different manufacturers use similar processes (Fig. 1A), there are variations in the final product, and produced from different levels of FG also have differences.

During fermentation, microorganisms consume the nutrients in FG, producing ethanol and volatile compounds (Tan et al. 2019). At the start of fermentation, FG contains a substantial amount of starch, over 20% of the weight of FG (Tan et al. 2019). Starch has strong water absorption properties, which decrease as the starch is consumed, allowing water to seep to the bottom of the pit and form *Huangshui* (Ren et al. 2024). Concurrently, *Huangshui* contains a complex mixture of substances, besides the water derived from the FG, it also includes other substances, such as reducing sugars and lactic acid also migrate to the pit’s bottom. These changes lead to differences between the FG above the *Huangshui* line (FG-A) and below the *Huangshui* line (FG-B) (Ren et al. 2024). Additionally, based on production experience and existing literature, *Baijiu* derived from FG-B is often considered higher quality than that from FG-A (Tan et al. 2019). This is likely due to the differences in fermentation conditions between FG-A and FG-B, although this has not been extensively documented in previous reports.

Fermentation is a crucial step in SFB production, involving the active participation of microorganisms from *Daqu*, pit mud, and the surrounding environment, these microorganisms contribute to the development of SFB’s unique flavor (HongSun, Li-Juan Chai, Guan-Yu Fang, et al., 2022; Y. Xu, Wang, Liu, Li, Zhang, Li, et al., 2021; M. Zhu, Zheng, Xie, Zhao, Qiao, Huang, et al., 2022). Research on FG derived from various raw materials has shown that differences in microbial community succession can lead to variations in SFB flavor during processing (Tan et al. 2019). Studies on microbial community succession in FG and the *Huangshui* line indicate that microorganisms from pit mud play a significant role in forming organic acids, suggesting that these microorganisms may contribute to the differences in fermentation conditions between FG-B and FG-A (Gao et al. 2020). These findings collectively highlight that FG in SFB fermentation varies over time and across different layers. However, there has been no research specifically examining the dynamic changes between FG-A and FG-B during SFB fermentation. Investigating the differences in the quality of *Baijiu* produced from FG-A and FG-B, along with their fermentation parameters, microbial structure, and aroma components, could provide valuable insights into these variations.

In the present study, a specialized in situ sampler was employed to collect FG samples throughout the SFB fermentation process. The microbial community structure of these FG samples was analyzed using high-throughput sequencing on an Illumina platform. Moreover, volatile compounds in the samples were detected using headspace solid-phase micro-extraction combined with gas chromatography-mass spectrometry (HS-SPME-GC-MS). Fermentation parameters-including temperature, acidity, starch content, alcohol concentration, and reducing sugars were measured and analyzed alongside the microbial sequencing data to assess microbial succession in FG during fermentation. The data obtained from this study aim to improve the understanding of the differences between FG-A and FG-B in SFB fermentation, which is beneficial for optimizing the production process and improving the quality of SFB.

## Materials and methods

### Experimental design and sampling method

The experiment was conducted at a renowned *Strong-flavor* distillery in Yibin City, Sichuan Province, China. To investigate the differences between FG-A and FG-B during the fermentation process, three rectangular fermentation pits were used. Each pit was filled with the same grain materials (sorghum, corn, wheat, rice, and glutinous rice), *Medium Temperature Daqu*, and production mix (Fig. 1B). The fermentation period was set to 70 days, which is within the normal range of 60–70 days. Based on the fermentation time, samples of FG-A and FG-B from the same cellar were collected. FG-A samples were collected from one pit at 1, 7, 14, 21, 28, 35, 42, 49, 56, 63, and 70 days after the start of fermentation. FG-B samples were collected at 7, 14, 21, 28, 35, 42, 49, 56, 63, and 70days, according to the timing of *Huangshui* formation (Fig. 1B). There were three sampling points in FG-B, and only the sample from the layer at the height of *Huangshui* was taken, and then all the samples were mixed together to form a single sample. All samples from each pit were mixed before analysis, three pits cellar were selected as parallel samples in each time point. In total, 99 FG-A samples and 90 FG-B samples were collected.

### Fermentation parameters analysis

Six standard fermentation parameters were analyzed: temperature, starch content, reducing sugar content, acidity, alcohol content, and the height of *Huangshui*. Acidity was measured by titration with 0.1 mol/L NaOH, using phenolphthalein as an indicator with a pH endpoint of 8.2. Total reducing sugar and starch content were determined using the methods described in previous studies (Bravo et al. 1998; Miller & G., 1959). Alcohol content was measured with an alcoholmeter following distillation. Temperature was monitored with a temperature sensor (FJT-P3-L, Wuhan Fenjin, China), which was fixed in position within the pit. The height of *Huangshui* was measured using custom-made testing equipment. The data for FG-A and FG-B were analyzed and compared using principal component analysis (PCA) with SIMCA 13.0 software.

### Volatile compounds analysis

Volatile compounds in FG-A and FG-B were extracted using headspace solid-phase microextraction (HS-SPME) and analyzed by gas chromatography-mass spectrometry (GC-MS) with an Agilent 8890 GC and 7250(Q-TOF)Mass spectrometer (Agilent Technologies, CA). The extraction procedure followed the methods outlined by previous reports (Deng et al. 2023; Yang, Fu, He, Zhang, Chai, Shi, et al., 2024). For each sample, 50 µL of n-butyl acetate was added as an internal standard. SPME was performed using a fiber coated with 50/30 µm divinylbenzene/carboxen on polydimethylsiloxane (Supelco, Bellefonte, PA, USA), based on the methods described by Gao et al. (Gao et al. 2014)and Yu et al(Yu, Li, Wan, Song, Zhang, Raza, et al., 2023). The GC conditions were as follows: helium was used as the carrier gas at a flow rate of 1 mL/min, with separations conducted using an Agilent DB-Wax column (60 m × 0.25 mm × 0.25 μm). Detection parameters were set according to Deng et al(Deng et al. 2023). All samples were analyzed in triplicate. To identify and categorize the volatile compounds, mass spectra were compared with the NIST 17a mass spectral library (National Institute of Standards and Technology, USA, https://www.nist.gov/). Lactic acid and acetic acid were quantified using liquid chromatography (LC) with a 1260 Infinity system (Agilent Technologies, CA). Solvent A comprised 20 mmol/L ammonium acetate and 20 mmol/L acetic acid, while solvent B was 100% acetonitrile. The LC gradient method followed Song et al(Song, Park, Kim, Jo, Kim, Theberge, et al., 2020). The concentrations of individual volatile compounds in FG-A and FG-B were reported in mg/kg. Peaks of ethanol were excluded, and compounds with concentrations below 0.1 mg/L or with similarity matches below 70% were filtered out. Data were analyzed using partial least squares projection to latent structure discriminant analysis (PLS-DA) with SIMCA 13.0 software (Umetrics, Sweden). A heatmap was generated using RStudio software to illustrate changes in volatile compound content.

### Microbial community structure analysis

FG samples were pretreated following a previously established method. Total DNA was extracted using Cetyltrimethyl Ammonium Bromide (CTAB) extraction method according to the manufacturer’s instructions(Wang et al. 2020). The extracted DNA was analyzed by electrophoresis on a 0.6% (w/v) agarose gel and by spectrophotometry (optical density ratio at 260 nm/280 nm). All DNA samples were stored at -80 °C. For bacterial analysis, the V3/V4 region of the 16 S rRNA gene was amplified using PCR with forward primer 338 F (5’-ACTCCTACGGGAGGCAGCA-3’) and reverse primer 806R (5’-GGACTACHVGGGTWTCTAAT-3’). For fungal analysis, the internal transcribed spacer (ITS) region was amplified using primers ITS1F (5’-CTTGGTCATTTAGAGGAAGTAA-3’) and ITS2R (5’-GCTGCGTTCTTCATCGATGC-3’). PCR was conducted in triplicate in a 20 µL reaction mixture using a MyCycler Thermal Cycler (Bio-Rad, USA), with amplification conditions as described by Deng et al(Deng et al. 2023). The amplified gene sequences were then sequenced using the Illumina MiSeq PE300 and NovaSeq PE250 platforms (Illumina, San Diego, USA) at Meiji Bio-Pharm Technology Co. Ltd. (Shanghai, China). High-throughput sequencing analysis methods were detailed in our previous work (Wang et al. 2020). The raw sequence reads have been deposited in the NCBI Sequence Read Archive (SRA) under accession numbers PRJNA720603 and PRJNA720773.

### Biomass analysis

Real-time PCR technology was used to analyze microbial biomass in FG-A and FG-B. A standard curve was constructed for the same primers used for bacterial (338 F/806R) and fungal (ITS1F/ITS2R) sequences. The absolute copy numbers of the 16 S rRNA and ITS genes in FG samples (with DNA from each batch of parallel samples pooled) were quantified using absolute qPCR on a PicoReal real-time PCR system (Applied Biosystems, ABI7300, USA). The qPCR protocol followed the method outlined by Du et al(Du et al. 2020). Each sample was tested in triplicate. Data visualization was performed using Origin 8, and significant differences between groups were analyzed with SPSS 24.0 software (IBM, USA).

### Statistical analysis

Changes in fermentation parameters were visualized using Origin 2018 (OriginLab, USA). Redundancy Analysis (RDA) was performed with Canoco 5 (http://www.canoco.com) to analyze relationships between fermentation parameters, microbial communities, and flavors. Correlation analysis was conducted using RStudio software. A network was created with Cytoscape to categorize and visualize the correlations between microbes and volatile compounds (Saito, Smoot, Ono, Ruscheinski, Wang, Lotia, et al., 2012). The function of the 16 S rRNA sequences was predicted using PICRUSt2 (Douglas, Maffei, Zaneveld, Yurgel, Brown, Taylor, et al., 2020).

## Results

### Changes in fermentation kinetic parameters during SFB fermentation

**Fig. 2:**
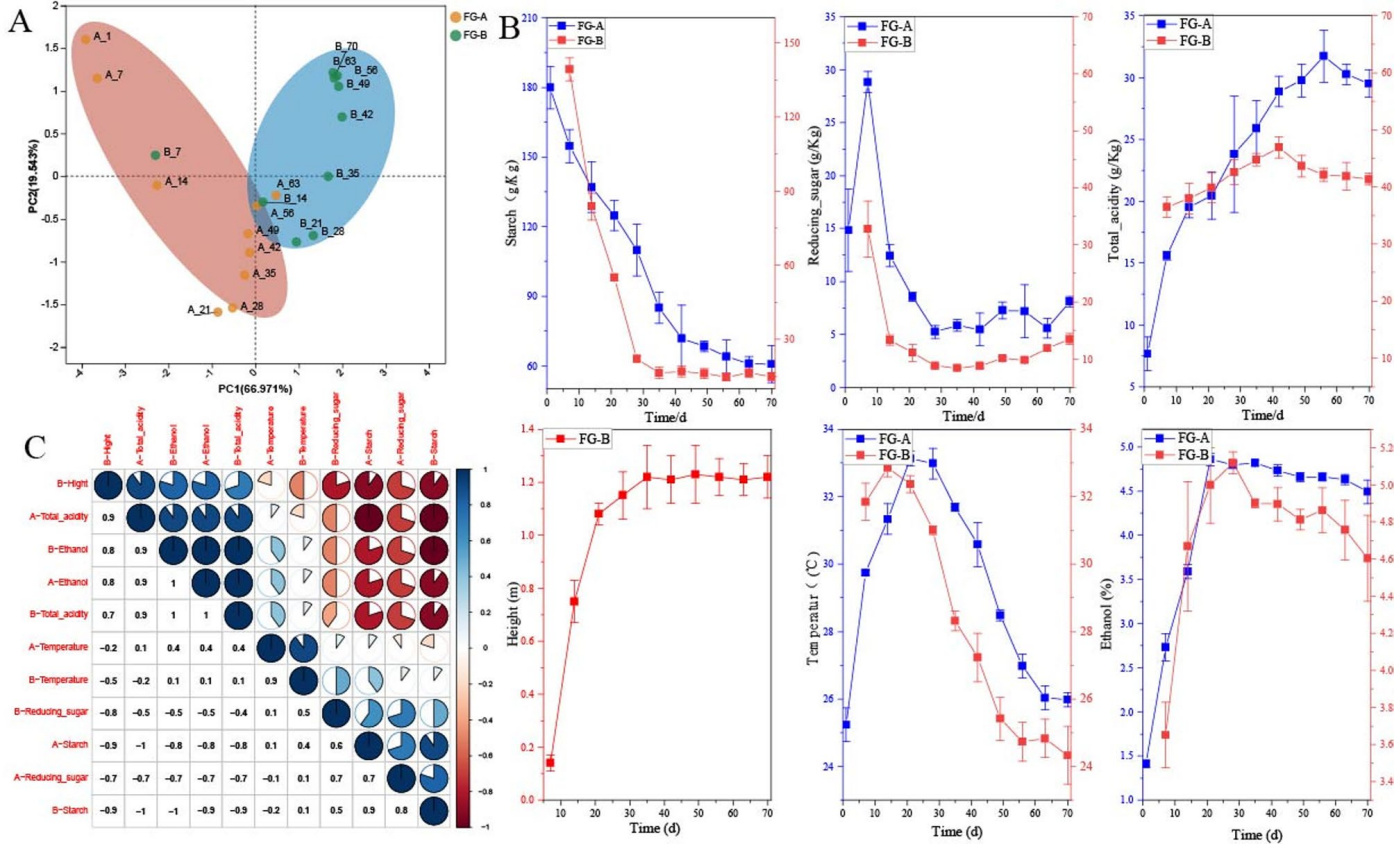
Changes in fermentation parameters. (**A**) Stages division according to principal component analysis (PCA) based on the dissimilarity matrix of fermentation parameters. (**B**) Correlation analysis of fermentation parameters. (**C**) Change trends in fermentation parameters during *NongXiangXing Baijiu* (SFB) fermentation

Fermentation parameters are crucial for monitoring the SFB fermentation process. In this study, six key parameters were measured: starch content, reducing sugar content, ethanol concentration, total acidity, temperature, and the height of the *Huangshui* line. Principal Component Analysis (PCA) was used to analyze the data, with the PCA score plots shown in Fig. 2A. The first principal component (PC1) explained 66.1% of the variance, while the second principal component (PC2) accounted for 28.2% of the variance, facilitating effective data interpretation.

Initially, before 23 days of fermentation process, FG-A and FG-B samples did not exhibit clear separation in the PCA score plots. However, differences between samples became apparent on day 23 after fermentation. PCA results indicated significant differences in fermentation parameters between FG-A and FG-B in the later stages of fermentation. Although FG-A and FG-B samples showed similar trends throughout fermentation, their parameter values differed (Fig. 2B).

In the early stages of fermentation, parameters changed rapidly: starch and reducing sugar contents decreased, while acidity, ethanol content, temperature, and the height of the *Huangshui* line increased. In the later stages, changes in parameters were less pronounced, with temperature showing a substantial decrease. The height of the *Huangshui* line and the contents of reducing sugar and starch remained relatively stable, while acidity and ethanol content in FG-A gradually decreased. Significant positive correlations were found between the height of the *Huangshui* line, ethanol, acidity, and starch and reducing sugar contents (*P* < 0.05) (Fig. 2C). Starch consumption and the formation of *Huangshui* are gradual processes. *Huangshui* did not form during the initial 10–15 days of fermentation. As fermentation progressed, the height of *Huangshui* increased, reaching approximately 1.1 m after 30 days.

### Changes in volatile compounds in FG during SFB fermentation

**Fig. 3:**
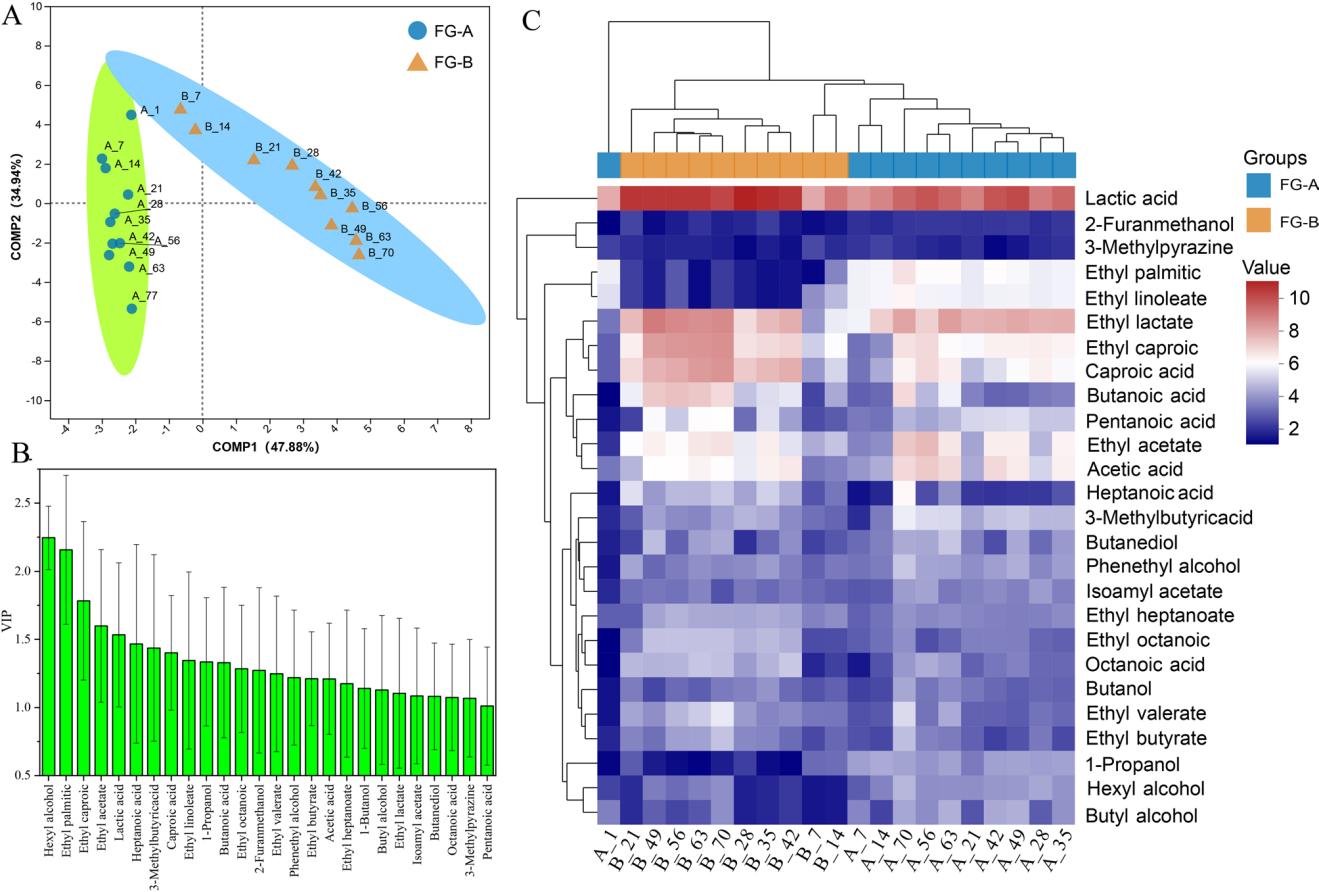
Changes in volatile compounds. (**A**) Partial least squares-discrimination analysis (PLS-DA) plots of volatile compounds of fermented grains in *NongXiangXing Baijiu* (SFB) above (FG-A) and below (FG-B) the *Huangshui* line. (**B**) Variable importance in projection values of the twenty-six markers of volatile compounds. (**C**) Heatmap of the twenty-six markers of volatile compounds

A total of 153 volatile compounds were detected in FG during the fermentation of SFB, including 45 esters, 24 acids, 26 alcohols, 12 ketones, 10 aldehydes, 10 alkenes, 7 furans, and 19 other compounds. Specifically, 133 volatile compounds were identified in FG-A, while 136 were found in FG-B, with 116 compounds present in both sample groups. Partial Least Squares Discriminant Analysis (PLS-DA) was used to analyze the volatile compound data, and the PLS-DA score plots are shown in Fig. 3A. R2X, R2Y, and Q2 in the PLS-DA model were 0.819, 0.955, and 0.828, respectively. FG-A and FG-B samples were clearly separated in the PLS-DA score plots. Variable Importance in Projection (VIP) scores greater than 1 highlighted differences in volatile compounds between FG-A and FG-B. Twenty-six volatile compounds were identified as potential markers of aroma quality (Fig. 3B). These included: 10 esters (ethyl palmitic, ethyl linoleate, ethyl caproic, ethyl acetate, ethyl lactate, ethyl octanoic, ethyl heptanoate, ethyl valerate, isoamyl acetate, and ethyl butyrate), 7 acids (lactic acid, caproic acid, heptanoic acid, octanoic acid, pentanoic acid, acetic acid, and butanoic acid), 7 alcohols (1-propanol, butanediol, phenethyl alcohol, 1-propanol, butyl alcohol, hexyl alcohol, and 2-furanmethanol), and 2 other volatile compounds (3-methylpyrazine and 3-methylbutane).

To normalize the data, a logarithmic scale (log^2(x)^) and unit variance scaling were applied to all volatile compound marker values. A heatmap and hierarchical cluster analysis (HCA) were then used to visualize the content of these volatile compounds in the samples (Fig. 3C). In the early stages of fermentation, the volatile compound markers in FG-A and FG-B were similar. However, in the later stages, significant differences emerged. Specifically, the contents of certain volatile compounds in FG-B were significantly higher than those in FG-A (*P* < 0.05). This included all esters and acids, except for ethyl palmitate and ethyl linoleate, as well as short-chain fatty acids (SCFAs) and their esters, such as butyric acid, caproic acid, ethyl butyrate and ethyl caproate, which warrant further investigation. Conversely, FG-A had significantly higher levels of other volatile compound markers compared to FG-B (*P* < 0.05), including six alcohols, several furans, and pyrazines. Thus, the differences in volatile compounds between FG-A and FG-B were more pronounced in the later stages of fermentation, with SCFAs and their esters being particularly important markers of these differences.

### Microbial community succession during SFB fermentation

High-throughput sequencing was used to analyze the microbial community structures in FG samples. After quality control, a total of 1,506,184 high-quality reads were obtained from ITS sequencing, while 1,344,819 high-quality reads were acquired from sequencing the V3-V4 region of the 16 S rRNA genes (using the primer pair 338 F-806R) from 36 FG-A samples and 30 FG-B samples. For fungal sequences, the average number of reads per sample was 68,463, ranging from 51,224 to 74,917. In contrast, bacterial sequences yielded an average of 61,128 reads per sample, with a range of 42,001 to 74,317 reads. Considering OTUs with more than 5 sequences as valid, a total of 885 OTUs were identified in the ITS sequences, representing 271 genera and 8 phyla. In the 16 S rRNA sequences, 3,432 OTUs were identified, corresponding to 1,018 genera and 40 phyla.

**Fig. 4:**
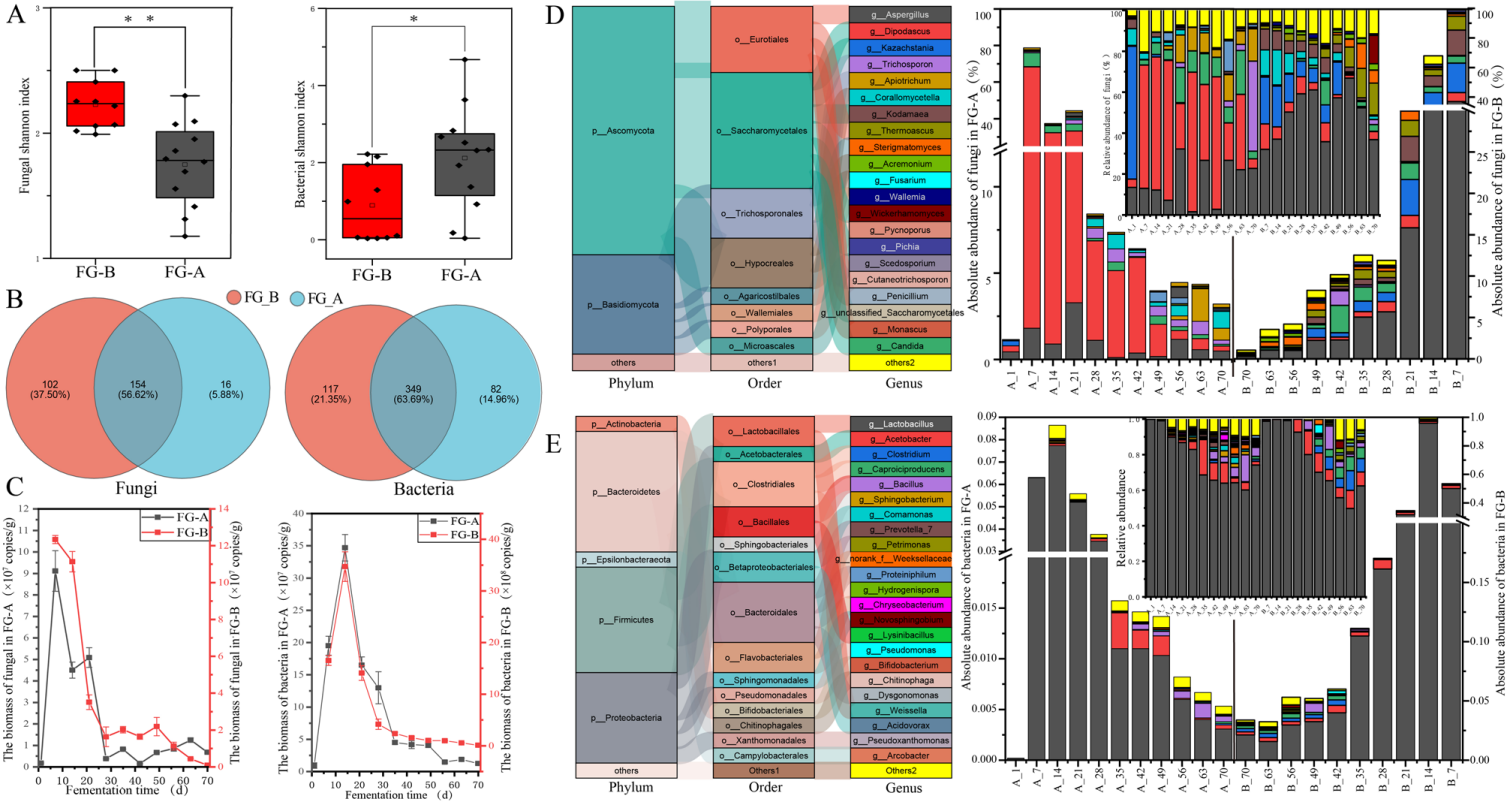
Microbial diversity and succession of fermented grains in *NongXiangXing Baijiu* (SFB) above (FG-A) and below (FG-B) the *Huangshui* line. (**A**) Venn diagrams of microbial genera in FG-A and FG-B (**p < 0.01, as determined by one-way ANOVA). (**B**) Box plots showing Shannon index values for fungi (right) and bacteria (left) (0.01< *p < 0.05; **p < 0.01, as determined by one-way ANOVA). (**C**) Biomass of bacteria (left) and fungi (right). (**D**) The fungal community structure at the genus level. (**E**) The bacterial community structure at the genus level

Based on the Shannon index, the fungal community diversity was significantly higher in FG-B samples compared to FG-A samples (*P* < 0.01). Conversely, bacterial community diversity was significantly higher in FG-A samples compared to FG-B samples (*P* < 0.05) (Fig. 4A). Regarding biomass during SFB fermentation, bacteria had a higher biomass than fungi. Both bacterial and fungal biomasses showed similar trends between FG-A and FG-B: they increased initially, then decreased, and finally stabilized after 40 days of fermentation. Specifically, bacterial biomass in FG-B was significantly higher than that in FG-A (*P* < 0.05). However, no significant differences in fungal biomass were observed between the samples (Fig. 4A).

Subsequently, the microbial community structure of the two sample groups was analyzed to understand the differences in microbial succession patterns during SFB fermentation. A total of 3 fungal phyla and 282 genera were identified. Among these, 266 genera were found in FG-B samples, while 169 genera were found in FG-A samples. The number of fungal genera in FG-B was significantly higher than in FG-A (*P* < 0.01) (Fig. 4B). Bar plots illustrated changes in the dominant phyla and genera, showing the relative abundance of microorganisms during fermentation (Fig. 4C and D). *Ascomycota* and *Basidiomycota* were the dominant phyla, with their relative abundances showing similar trends in FG-A and FG-B samples. Specifically, the relative abundance of *Ascomycota* gradually decreased, while the relative abundance of *Basidiomycota* gradually increased throughout the fermentation process.

**Fig. 5:**
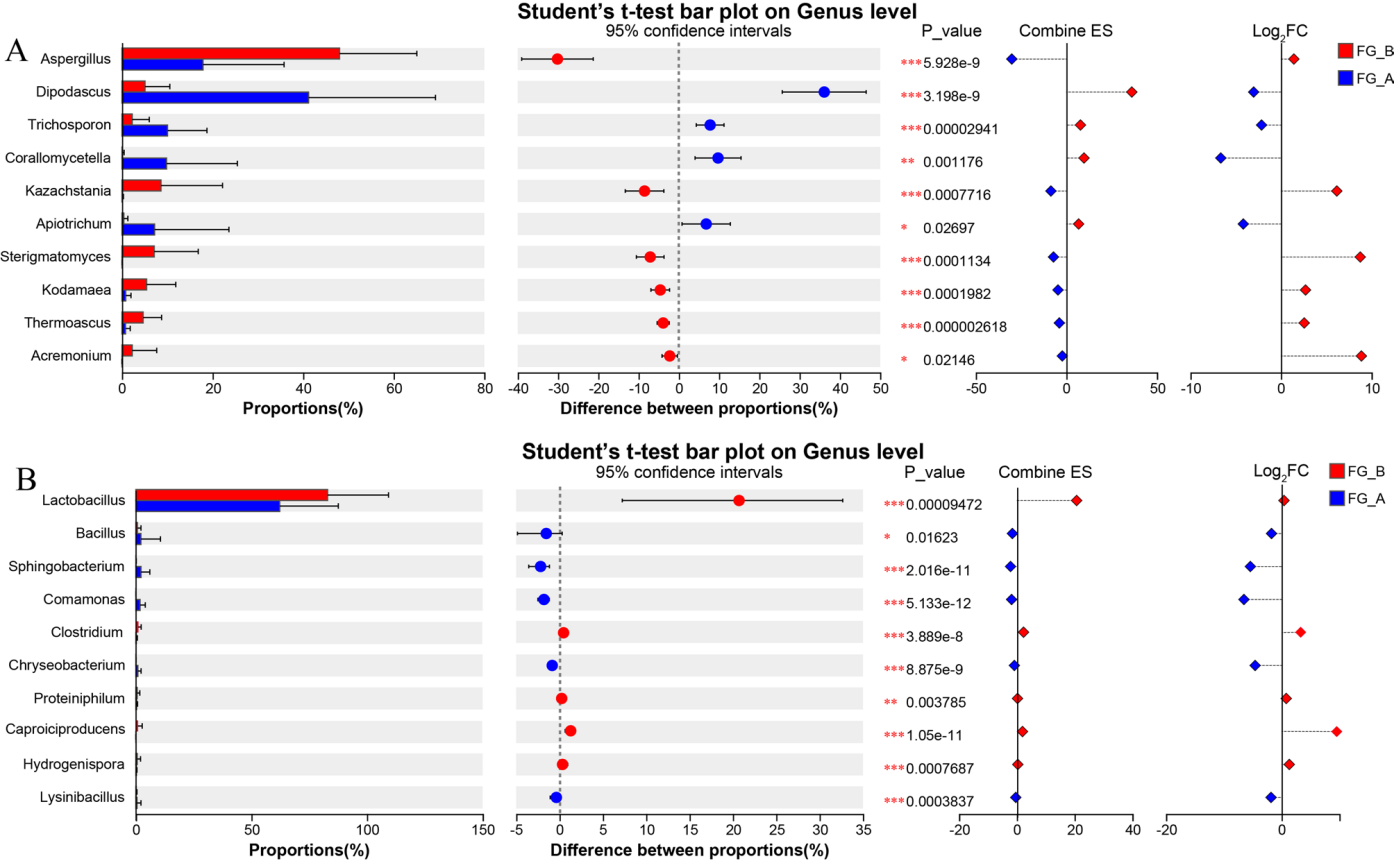
Distinction between fungal and bacterial genera in FG-A and FG-B. (**A**) Different fungi genera based on Student’s t-test; (**B**) Different bacterial genera based on Student’s t-test (0.01< *p < 0.05; 0.001< **p < 0.01; ***p < 0.001)

The top 20 genera (relative abundance > 2%) were identified. In FG-B, *Dipodascus* was the most abundant genus, followed by *Kazachstania*. In contrast, *Aspergillus* was the most abundant genus in FG-A, followed by *Dipodascus*. In FG-B, the relative abundance of *Kazachstania*, *Kodamaea*, and *Thermoascus* gradually decreased, while *Sterigmatomyces*, *Apiotrichum*, and *Pycnoporus* increased. Conversely, in FG-A, the relative abundance of *Dipodascus* gradually decreased, while *Trichosporon*, *Corallomycetella*, and *Pichia* increased. Overall, significant differences were observed in 8 fungal genera between FG-A and FG-B samples (99% confidence intervals). The abundances of *Dipodascus*, *Trichosporon*, and *Corallomycetella* were significantly higher in FG-A compared to FG-B, whereas the abundances of *Aspergillus*, *Kazachstania*, *Sterigmatomyces*, *Kodamaea*, and *Thermoascus* were significantly higher in FG-B (*P <* 0.01) (Fig. 5A).

Regarding bacterial phyla, *Firmicutes*, *Proteobacteria*, *Bacteroidetes*, *Actinobacteria*, *Patescibacteria*, and *Chloroflexi* were the most abundant, each with a relative abundance greater than 2%. In FG-A, the relative abundance of *Firmicutes* gradually decreased during SFB fermentation, while *Proteobacteria*, *Bacteroidetes*, *Actinobacteria*, *Patescibacteria*, and *Chloroflexi* increased. For FG-B, *Firmicutes* dominated, accounting for approximately 98% of the bacterial community from days 16 to 41 but decreased to 85% after 47 days of fermentation. A significant increase in *Proteobacteria* and *Bacteroidetes* was observed in FG-B after 47 days (Fig. 4D).

A total of 1,028 bacterial genera were identified, with 606 genera found in FG-B and 981 in FG-A. The number of bacterial genera in FG-A was significantly higher than in FG-B (*P* < 0.01) (Fig. 5B). Among the 19 dominant bacterial genera (relative abundance > 2%), notable ones included *Lactobacillus*, *Acetobacter*, *Clostridium*, *Caproiciproducens*, and *Bacillus* (Fig. 4D). In FG-A, *Lactobacillus* was the most abundant genus, with a relative abundance greater than 90% during early fermentation stages. As fermentation progressed, *Lactobacillus* abundance decreased, while *Acetobacter*, *Bacillus*, *Comamonas*, and *Sphingobacterium* increased. In FG-B, *Lactobacillus* remained the dominant genus, accounting for over 95% of the bacterial community from days 16 to 41. However, after 47 days, the relative abundances of *Acetobacter*, *Caproiciproducens*, *Clostridium*, *Prevotella_7*, *Hydrogenispora*, and *Petrimonas* increased. Significant differences were found in 9 bacterial genera between FG-A and FG-B (99% confidence intervals). *Lactobacillus*, *Clostridium*, *Proteiniphilum*, *Hydrogenispora*, and *Caproiciproducens* were significantly more abundant in FG-B compared to FG-A, while the remaining 4 genera were more abundant in FG-A (*P* < 0.01) (Fig. 5B). Overall, these results indicate significant differences in microbial community structures between FG-A and FG-B during SFB fermentation.

### Effects of microorganisms on FG

**Fig. 6:**
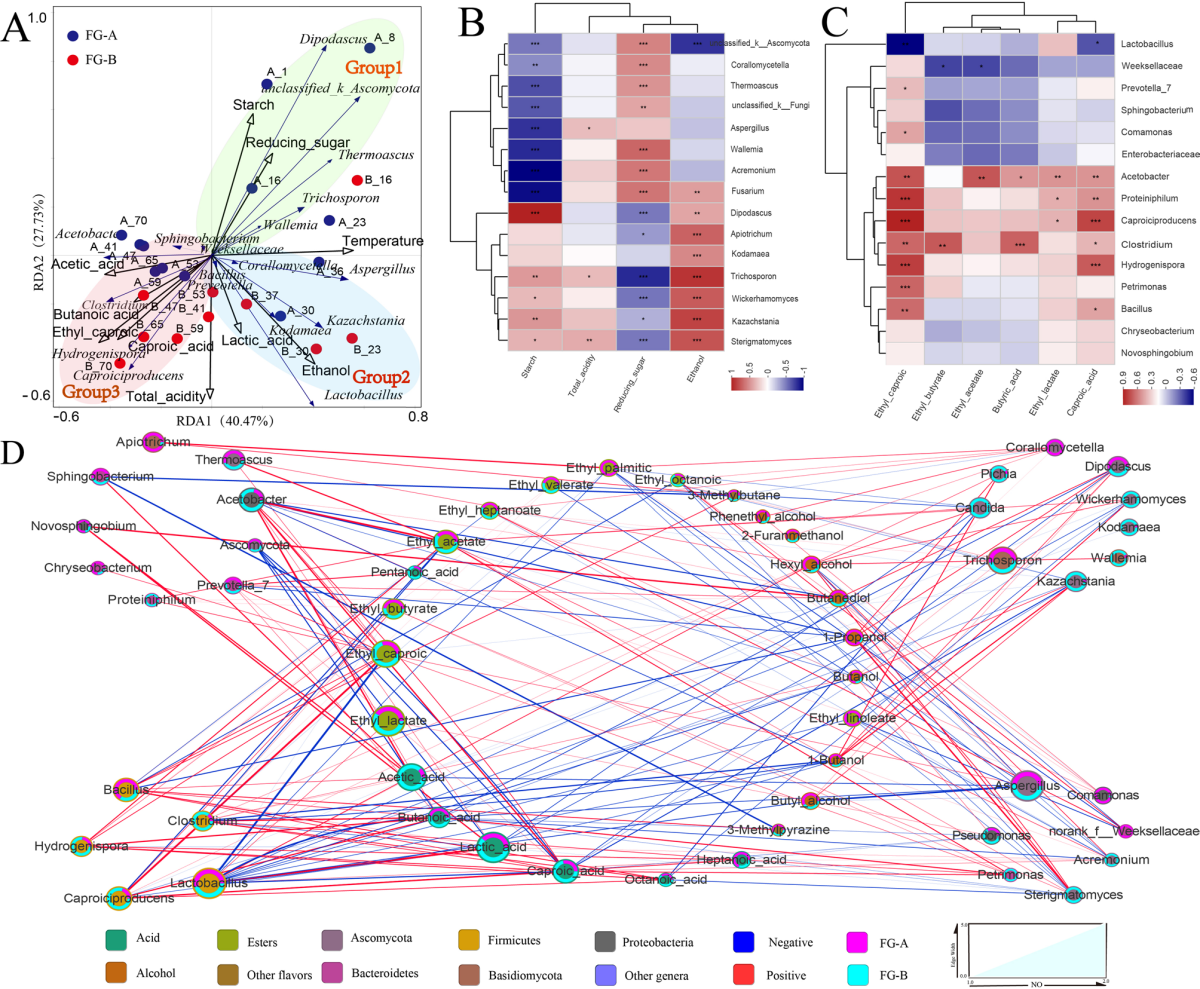
The relationship among fermentation parameters, flavor and microbial communities in in *NongXiangXing Baijiu* (*SFB*) above (FG-A) and below (FG-B) the *Huangshui* line. (**A**) Redundancy analysis (RDA) of microbial community composition and fermentation parameters. (**B**) Correlation analysis of major fungal genera and fermentation parameters (0.01< *p < 0.05; 0.001< **p < 0.01; ***p < 0.001, as determined by one-way ANOVA). (**C**) Correlation analysis of major bacterial genera and short-chain fatty acids (SCFAs) and their esters(0.01< *p < 0.05; 0.001< **p < 0.01; ***p < 0.001, as determined by one-way ANOVA). (**D**). Network analysis of microorganisms and flavor

Redundancy Analysis (RDA) was employed to elucidate the relationship between microbial succession and fermentation stages in SFB production. The RDA results indicated that the two principal axes accounted for 68.2% of the total variance in the microbial community data. The RDA score plots identified three distinct sections labeled Group1, Group2, and Group3 (Fig. 6A), each corresponding to different fermentation stages. In the early-stage fermentation, characterized by high starch content, Group1 reflected this phase where starch was converted into reducing sugars by the enzymatic activity of molds from *Daqu*. Molds such as unclassified_k_*Ascomycota*, *Aspergillus*, and *Wallemia* showed significant negative correlations with starch content (*P* < 0.05), indicating their role in starch hydrolysis (Fig. 6B). Reducing sugars, essential for microbial growth, serve as substrates for various species, including yeasts, which are crucial for ethanol production. Yeasts demonstrated significant negative correlations with reducing sugar content, while ethanol content showed significant positive correlations with yeast (*P* < 0.05) (Fig. 6B). Additionally, *Lactobacillus* utilized reducing sugars for lactic acid production, corresponding to Group2. Group1 and Group2 are associated with the early stages of fermentation, during which ethanol and lactic acid accumulate. Group3 represents the late-stage fermentation, where the production of short-chain fatty acids (SCFAs) and their esters becomes prominent. *Acetobacter*, *Clostridium*, and *Caproiciproducens* were identified as key functional microorganisms in this stage, contributing significantly to SCFA production.

To elucidate the influence of microbial species on the differences in volatile compounds between FG-A and FG-B, correlation analysis (Fig. 6C) and correlation network analysis (Fig. 6D) were performed. Yeasts exhibited positive correlations with alcohol content and negative correlations with acid content. This aligns with their role as primary producers of alcohols during SFB fermentation, where excessive acid production inhibits their growth and metabolism, as supported by both the correlation analysis (Fig. 6B) and variations in fungal biomass (Fig. 4C).

Additionally, several bacterial genera showed significant correlations with the contents of SCFAs and their esters, including *Acetobacter*, *Bacillus*, *Hydrogenispora*, *Caproiciproducens*, and *Clostridium*. Specifically, *Acetobacter* and *Clostridium* showed significant positive correlations (*P* < 0.05) with caproic acid and butyric acid contents. *Caproiciproducens* and *Hydrogenispora* showed highly significant positive correlations (*P* < 0.01) with caproic acid and ethyl caproic contents and *Lactobacillus* showed significant negative correlations with them (*P* < 0.05).

### Exploring differences in SCFAs and SCFAEs in FG

**Fig. 7:**
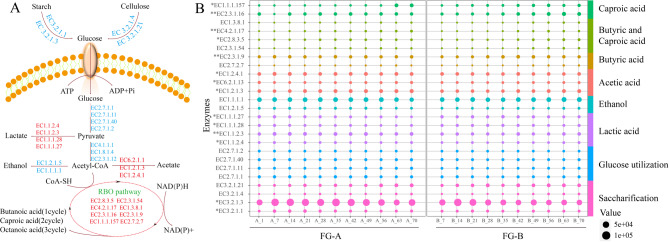
Potential functions of the microbial community of fermented grains in the production of short-chain fatty acids (SCFAs). (**A**) SCFAs Metabolic pathways. (**B**) Differences in the abundance of enzymes involved in SCFAs metabolism in *NongXiangXing Baijiu* (SFB) above (FG-A) and below (FG-B) the *Huangshui* line. (0.01< *p < 0.05; **p < 0.01, as determined by one-way ANOVA)

To further understand the effects of microorganisms on volatile compound metabolism, particularly SCFA production, we employed PICRUSt2 to analyze microbial sequencing data and construct microbial metabolic pathways related to SCFA production in FG (Fig. 7A). The abundance of key enzymes in these pathways was visualized using bubble charts (Fig. 7B).

Starch and cellulose in the FG are first broken down into reducing sugars. This process is facilitated by hydrolytic enzymes from *Daqu*, particularly amylase (EC 3.2.1.1, EC 3.2.1.3) and cellulase (EC 3.2.1.4, EC 3.2.1.21). The content of amylase was significantly higher in FG-A and FG-B during the early stages of fermentation compared to the late stages, likely due to the higher biomass of molds present initially (*P* < 0.05).

Microorganisms convert glucose to pyruvate through the Embden-Meyerhof-Parnas (EMP) pathway. This pyruvate can then be anaerobically metabolized into lactic acid and ethanol. Lactobacillus and yeast are the primary microorganisms responsible for producing these substances (Tan et al. 2019). The abundance of lactate dehydrogenases, which are crucial for converting pyruvate into lactic acid, was significantly different between FG-A and FG-B, with FG-B showing higher levels (*P* < 0.05). This explains the higher lactic acid content observed in FG-B during fermentation.

Acetic acid is produced from pyruvate via oxidative dehydrogenation. In FG-B, the abundance of aldehyde dehydrogenase (EC 1.2.1.3) and acetate thiokinase (EC 6.2.1.1) was higher compared to FG-A, whereas pyruvate dehydrogenase (EC 1.2.4.1) was more abundant in FG-A. This differential enzyme abundance impacts the formation and levels of acetic acid in the samples.

SCFAs are produced by reversing the β-oxidation cycle, utilizing ethanol and lactic acid as electron donors and acetyl-CoA as an electron acceptor. In late-stage fermentation, enzymes involved in SCFA production are more abundant. The abundance of five enzymes was significantly different (*P* < 0.05). In particular, the abundance of acetyl-CoA C-acetyltransferase (EC 2.3.1.9) and acetyl-CoA C-acyltransferase (EC 2.3.1.16), which are critical for chain elongation in SCFA production, was significantly higher in FG-B compared to FG-A (*P* < 0.01).

## Discussion

FG serves as the medium for microbial growth during SFB fermentation, and changes in fermentation parameters reflect the evolving environment within FG, thereby indicating the progress of the fermentation process. By analyzing specific fermentation kinetic parameters, the fermentation state of FG can be inferred. Based on these parameters, SFB fermentation can be divided into two distinct stages: (i) the early stage, encompassing the first 23 days, and (ii) the late stage, extending from day 24 onwards, which aligns with previous studies (Huang et al. 2024; Tan et al. 2019). During the early fermentation stage, the starch content in FG was approximately 180 g/kg. This starch was subsequently converted into reducing sugars by saccharifying enzymes produced by *Daqu* bacteria and molds. These reducing sugars were then utilized by microorganisms to produce ethanol and lactic acid (Ren et al. 2024). The reduction in starch content led to decreased water absorption, causing water and other liquids to accumulate at the bottom of the pit, forming *Huangshui*, and the composition and formation process of *Huangshui* have been reported in previous studies (Z. Ren, Chen, Q., Tang, T., HuanRen et al. 2024a, b, c). The formation of *Huangshui* increased the moisture level in FG-B and transformed it into a semi-solid state, facilitating material exchange within FG-B and promoting the migration of microorganisms from the pit mud. Additionally, *Huangshui* formation allowed for the percolation of certain substances from FG-A to FG-B, resulting in higher levels of reducing sugars, starch, ethanol, and total acidity in FG-B (Gao et al. 2020). Consequently, the formation of *Huangshui* created distinct fermentation conditions and nutrient compositions in the two layers of FG, which are crucial factors influencing differences in flavor and microbial community structure in SFB.

SFB fermentation involves a mixture of FG, *Daqu*, and grains, with various microorganisms-including those from *Daqu*, pit mud, and the environment-contributing to the flavor of SFB (Y. Li, Liu, Zhang, Liu, Qin, Shen, et al., 2022). At the outset of SFB fermentation, FG-A and FG-B exhibited similar volatile compounds. This similarity can be attributed to the fact that the original FG contained similar volatile compounds, such as ethyl palmitic and ethyl linoleate, whose concentrations remained relatively unchanged throughout the early stages of fermentation. However, their content decreased in FG-B due to dilution from *Huangshui*. In the early stages of fermentation, the volume of *Huangshui* was low, and both layers of FG were in a solid state. Consequently, the volatile compounds in *Huangshui* were derived from the upper layer of FG, resulting in similar volatile profiles for FG-A and FG-B during this period. As fermentation progressed and the volume of *Huangshui* increased, initial fermentation products accumulated in the lower layer. This led to a semi-solid state in FG-B after 30 days of fermentation.

The content of alcohols in FG-A was significantly higher than in FG-B, making alcohols a key differential volatile compound (*P* < 0.05). Alcohols are crucial in SFB production, as they contribute to the fragrant, mellow, and sweet sensory characteristics of *Baijiu* (Chen, Zhao, Chen, Zhang, Li, Zhao, et al., 2024). Additionally, FG-B had significantly higher levels of fatty acids and their esters compared to FG-A (*P* < 0.0), particularly lactic acid, acetic acid, butyric acid, and caproic acid. It is well-established that the main aroma components in SFB are four esters: ethyl caproate, ethyl lactate, ethyl butyrate, and ethyl acetate. The content and proportion of these esters are critical in determining the type and quality of SFB (Qian, Lu, Chai, Zhang, Li, Wang, et al., 2021). The observed differences in the levels of these fatty acids and esters between FG-A and FG-B reflect variations in aroma composition and the quality of the final *Baijiu* product. Therefore, the differences in fermentation status and nutrient composition between FG-A and FG-B likely explain the variations in volatile compounds, which in turn may influence the quality of the original *Baijiu*.

Microorganisms are the primary producers of flavor compounds in SFB fermentation. During the early stages of fermentation, fungal biomass was notably higher, especially in FG-A. Fungi contribute to the breakdown of starch in FG, resulting in the formation of reducing sugars, which are crucial substrates for the metabolism of ethanol by microorganisms (Q. Zhu, Chen, Peng, Zhang, Huang, Yang, et al., 2022). An increase in yeast biomass before 23 days of fermentation promoted ethanol production, yeast is not only the main metabolizing microorganism of ethanol, but also the main contributor of higher alcohols (Huang et al. 2024). The abundance of yeast in FG-A is higher than that of FG-B, especially Dipodascus, which is the absolute dominant fungal genus during the whole fermentation process, and many higher alcohols in FG-A are significantly higher than that of FG-B, which also indicates that yeast may be an important producer of higher alcohols in the fermentation process of SFB. Previous studies have shown that long-chain fatty acids may be derived more from lipid hydrolysis in feedstock than from microbial synthesis. However, there are also microorganisms in FG that can utilize other substances to produce long-chain fatty acids, such as *Trichosporon*, a basidiomycetous oleaginous yeast capable of converting glucose into grease (Yaguchi et al. 2017), may have been involved in the synthesis of long-chain fatty acid esters in FG. *Aspergillus*, known for producing amylase and esterase in *Baijiu* (Ma, Luo, Zhao, Qiao, Zheng, An, et al., 2022), was significantly more abundant in FG-B than in FG-A. This abundance likely explains the higher ester content in FG-B. In the late stages of fermentation, microbial diversity in fermented grains did not decrease, a decrease in microbial abundance was observed, likely due to elevated ethanol content and high acidity in FG. These conditions create an anaerobic environment in the pit, which inhibits further microbial growth.

In the process of SFB fermentation, significant differences were observed in bacterial biomass between FG-A and FG-B, although the trends in both were similar. The similarity in bacterial Shannon indexes suggests that FG requires extended periods to sustain high bacterial diversity, consistent with findings from other studies (Lee, Lee, Singh, Oh, Jeon, Ryu, et al., 2017). *Lactobacillus* is the most dominant bacteria, and the main producer of lactic acid during SFB fermentation (Du et al. 2020). When *Lactobacillus* dominated the bacterial community in the early stage, particularly in FG-B, leading to a peak in lactic acid content around 30 days. Lactic acid from FG-A follows *Huangshui* into the bottom and microbial fermentation may be a significant factor contributing to the higher lactate levels observed in FG-B compared to FG-A.

In late-stage fermentation, the abundance of *Acetobacter*, *Sphingobacterium*, and *Bacillus* increased in FG-A due to environmental conditions, indicating a shift in the bacterial community from lactic acid fermentation to acetic acid fermentation (Li et al. 2009). In contrast, FG-B saw an increase in *Clostridium*, *Acetobacter*, *Hydrogenispora*, and *Caproiciproducens*, suggesting a shift towards caproic acid fermentation typical of anaerobic sludge environments (Q. Wang, Zhang, Bao, Liang, Wu, Chen, et al., 2020). Interestingly, the contents of caproic acid and ethyl caproate in FG-B increased significantly in late-stage fermentation, and their contents were significantly higher than FG-A (*P* < 0.01), while acetic acid and ethyl acetate increased significantly in FG-A, and their contents were significantly higher than FG-B (*P* < 0.01).*Huangshui* line did not rise in late-stage fermentation, indicating that the substances in FG-A infiltrated into FG-B basically stopped following *Huangshui*, the distinct the main volatile compounds in FG-A and FG-B are closely related to the microbial community structure in SFB.

Previous studies have identified large populations of *Clostridium*, *Hydrogenispora*, and *Caproiciproducens* in pit mud, with *Caproiciproducens* alone accounting for up to 34.79% of the total bacterial species on the surface (Chai, Qian, Zhong, Zhang, Lu, Zhang, et al., 2021; Huilin Wang, Yang Gu, Weicheng Zhou, Dong Zhao, Zongwei Qiao, Jia Zheng, et al., 2021; Qian et al. 2021). The presence of these bacteria indicates an enhanced capacity for fatty acid metabolism, which may be transported to FG-B via *Huangshui*, leading to increased fatty acid production during late-stage fermentation (Ren et al. 2024). Thus, the environmental conditions and pit mud microorganisms significantly influence the microbial community structure in FG-B. These differences in microbial composition likely contribute to the observed variations in volatile components between FG-A and FG-B during late-stage fermentation. In particular, acid-producing bacteria play a crucial role in influencing the production of short-chain fatty acids (SCFAs) and their esters in SFB.

In early-stage fermentation, a significant accumulation of lactic acid and ethanol occurred in FG. Both ethanol and lactic acid serve as electron donors for the production of SCFAs. Critical enzymes in this process include acetyl-CoA C-acetyltransferase (EC 2.3.1.9) and acetyl-CoA C-acyltransferase (EC 2.3.1.16). Under anaerobic conditions, *Acetobacter* and *Clostridium* convert ethanol into acetic acid and butyric acid, with *Clostridium* being particularly essential for butyric acid production. These acids then serve as precursors for the formation of other acidic chains. *Caproiciproducens* is the primary bacterial species responsible for producing caproic acid during SFB fermentation. This species is involved in chain elongation via the β-oxidation cycle to synthesize caproic acid (H. Wang, Li, Wang, Tao, Lu, Zhu, et al., 2018), with *Hydrogenispora* also supporting caproic acid production.

Interestingly, despite lactic acid being an electron donor for SCFA production, *Lactobacillus* exhibited negative correlations with several SCFAs and their esters, including caproic acid, butyric acid, ethyl caproic acid, and ethyl butyrate. This may be attributed to the shift from lactic acid and acetic acid fermentation to caproic acid fermentation, which led to a decrease in *Lactobacillus* abundance and an increase in *Hydrogenispora*, *Caproiciproducens*, and *Clostridium*.

Significant differences were observed in the abundance of acid-producing microbial species between FG-A and FG-B during SFB fermentation. In particular, the absolute abundance of *Clostridium*, *Caproiciproducens*, and *Hydrogenispora* was higher in FG-B compared to FG-A. These microbial species contribute to SCFA production, similar to findings reported in resource recovery studies on caproic acid production (Han et al. 2018; Wang et al. 2020a, b). This microbial profile likely promotes the formation of SCFA esters, potentially explaining the higher content of volatile compounds in FG-B compared to FG-A. Overall, significant differences in the abundance of microorganisms and enzymes involved in SCFA production between FG-A and FG-B were observed. These differences help explain the variations in SCFA and ester content between the two sample types during SFB fermentation.

The levels and equilibrium of SCFAs and their esters directly impact SFB quality. Environmental conditions and microbial community structure differences between FG-A and FG-B significantly influence the content of SCFAs and their esters. These findings provide valuable insights into the production differences of original *Baijiu* between FG-A and FG-B and offer potential strategies for standardizing SFB production. For example, adjusting the *Huangshui* line height by modifying pit shape could increase the proportion of FG-B. Additionally, understanding the role of microorganisms in pit mud can guide the application of functional bacteria in *Baijiu* production. Reducing the differences between FG-A and FG-B could further enhance SFB quality. In this study, significant differences were observed between FG-A and FG-B in terms of microbial community structure in late-stage fermentation, which might be attributed to the differences in the microbial community of pit mud. Further studies will be conducted on the effects of pit mud microorganisms on the assembly and metabolism of fermented grains in SFB.

## Conclusions

In the present study, we characterized the microbial community structure of FG-A and FG-B in SFB fermentation and analyzed the associated fermentation parameters and volatile compounds. Significant differences were observed between FG-A and FG-B in terms of fermentation parameters, volatile compounds, and microbial community structure. These variations in fermentation parameters influenced the microbial community structure, which, in turn, affected the aroma quality of FG. Twenty-six discriminant volatile compounds were found between FG-A and FG-B, including 9 esters, 6 acids, 5 alcohols and 6 other volatile compounds. Specifically, differences in the content of short-chain fatty acids and their esters between FG-A and FG-B directly impacted the quality of the resulting SFB. Seventeen differential genera were observed between FG-A and FG-B, including 9 bacterial genera and 8 fungal genera. The difference of bacterial communities may be the main reason for the difference in the contents of short-chain fatty acids and their esters in FG-A and FG-B. These findings provide new insights into SFB production and offer a foundational understanding for better monitoring and optimization of the fermentation process.

## Data Availability

Not applicable.
